# Can Anterior Knee Pain Be Explained by Patella Position After Infrapatellar Tibia Intramedullary Nailing?

**DOI:** 10.7759/cureus.47334

**Published:** 2023-10-19

**Authors:** Batuhan Gencer, Alperen Yiğit, Can Çamoğlu, Ahmet Çulcu, Ozgur Dogan

**Affiliations:** 1 Department of Orthopaedics and Traumatology, Sancaktepe Şehit Prof. Dr. İlhan Varank Training and Research Hospital, İstanbul, TUR; 2 Department of Orthopaedics and Traumatology, Ankara Bilkent City Hospital, Ankara, TUR; 3 Department of Orthopaedics and Traumatology, Yüksekova State Hospital, Hakkâri, TUR

**Keywords:** patellar height disorder, patella-patellar tendon angle, anterior knee pain, intramedullary nailing of the tibia, tibial shaft fracture

## Abstract

Objective: The objective of this study is to investigate the postoperative position of the patella and its relationship with anterior knee pain in patients operated with infrapatellar reamed tibia intramedullary nailing (IMN).

Materials and methods: Patients who underwent tibia IMN between 2019 and 2022 and who had anterior knee pain in their postoperative follow-up at least two outpatient clinic controls with an interval of at least one month were examined. Patellar height indices (Insall-Salvati, Blackburne-Peel, Caton-Deschamps, and modified Insall-Salvati) and sagittal angulation (patella-patellar tendon angles) were measured on the lateral direct radiographs of the patients in semi-flexion. As a control group, measurements were made on the contralateral intact extremity radiographs of the same patients.

Results: There was no significant difference in patellar height indices between the fractured and intact sides in any of the patients (p = 0.588; p = 0.747; p = 0.446; p = 0.573, respectively). When the sagittal angulations were analyzed, a significant difference was found between the fractured and intact sides of the patients (p = 0.048), resulting in an approximate three-degree change.

Conclusion: Patellar sagittal balance has been identified as one of the contributing factors to the development of anterior knee pain following reamed tibial IMN. Further biomechanical and comprehensive clinical studies are needed on this subject.

## Introduction

Tibial intramedullary nailing (IMN) has been shown to achieve an 80% success rate in treating tibial shaft fractures [1]. IMN may be conducted through either the infrapatellar or suprapatellar approach [1,2]. In the case of the infrapatellar approach, various methods have been defined, such as the medial/lateral parapatellar approach, which maintains the patellar tendon, and the patellar tendon split approach, which dissects through the middle of the patellar tendon [2]. Among the complications associated with tibial intramedullary nailing, anterior knee pain stands out as one of the most prevalent complications with an incidence of 30%-50% of patients undergoing tibia IMN [1-5]. Anterior knee pain is frequently associated with the infrapatellar surgical approach, the proximal entry point of the nail, and the thickness and width of the patellar tendon [2].

Knee stability and extensor mechanism are significantly impacted by patellar height and the patellofemoral sagittal relationship [6-10]. Although many studies have examined the effect of patella position (height and sagittal angulation) on knee-related clinical conditions like patellofemoral instability, total knee arthroplasty, high tibial osteotomy, and meniscal tear, limited research exists on the correlation between patella position and anterior knee pain following tibial intramedullary nailing [9,11,12].

The present study aimed to investigate the relationship between the postoperative positioning of the patella concerning height and sagittal angulation as well as the incidence of anterior knee pain following tibial IMN.

## Materials and methods

Medical records of 57 patients who underwent IMN of the tibia between May 2019 and January 2022 were examined, with the approval of the ethics committee (Decision No: E1-22-2544, dated April 6, 2022). All participants aged 18 or over, who were able to move without pain before surgery, who underwent a minimum follow-up period of one year post-surgery, and who experienced ongoing anterior knee pain during at least two outpatient clinic appointments one month apart were eligible for inclusion in the study. Patients with tibial shaft fractures extending to the proximal or distal articular surface, those who received treatment with external fixator or plate osteosynthesis, those with polytrauma, those undergoing staged surgery, those with Gustillo-Anderson type 3 open fractures, those who reported anterior knee pain at postoperative follow-up, and those who declined to participate in the study were excluded. Type 3 fractures were not included in the study due to their distinctive follow-up and treatment mechanism in comparison to other types as well as the varied complication rates. A total of 57 patients received intramedullary nailing for fractures of the tibial shaft within the specified date range. Among them, considering the inclusion and exclusion criteria, 19 patients who persisted with associated anterior knee pain at least one month apart during their follow-up outpatient appointments were evaluated.

Surgical technique

All patients received spinal anesthesia without a tourniquet while lying in the supine position. Following the recommended literature [1,2,13], we followed an infrapatellar patellar tendon split approach and used a reamed intramedullary tibial nail. To prevent patellar tendon injury during surgery, all precautions were taken, including the use of a tendon protector and keeping the height of the nail at the entry site standard to avoid disruption of the joint and tendon. Those patients with concomitant lateral malleolus fractures underwent treatment using a traditional "J" incision over the lateral malleolus and a distal fibula anatomic plate or 1/3 tubular plate, depending on the fracture geometry, as recommended in the literature [14]. All surgical procedures were carried out by the same surgeon.

Post-surgery, patients started with weight-bearing as tolerated and gradually increased it according to tolerance. If a lateral malleolus fracture was fixed simultaneously, load initiation would be delayed for a maximum of one month. Postoperative follow-up of all patients was performed without plaster/splint, and active-passive range of motion and quadriceps exercises were started on the first postoperative day with the help of physiotherapists.

For patients with Gustillo-Anderson type 1 and 2 open fractures, reamed tibial intramedullary nails were used after antibiotherapy and washing in the emergency department [15]. Gustilo-Anderson grade III open fractures were not considered in the study as per the exclusion criteria.

Clinical and radiological examination

The study collected demographic data, the mechanism of injury (simple fall, fall from a height, or traffic accident), the type of open fracture (none, Gustillo-Anderson type 1 or 2), the location of the fracture (proximal 1/3, middle 1/3, or distal 1/3), and geometric fracture features (spiral, oblique, transverse, segmentary, or complex-irregular) of the patients. Subsequently, the patients were contacted by telephone, using the contact details provided, to attend the outpatient clinic. Lateral direct X-rays were taken with the knee flexed at 30° to assess the sagittal angulation and patellar height. A comparison of preoperative and postoperative measurements for changes would have been desirable, but such measurements were not feasible, given the sudden nature of the injuries sustained by trauma patients. Hence, notwithstanding controversy in the literature, it was assumed that the patient's anatomy was equivalent on both sides, and comparisons were made with the whole extremity on the opposite side [6,16]. Measurements were taken by both authors (CÇ and AY) independently using the hospital's imaging technology, SarusPACS. In three cases (15.79%) where the results were inconsistent according to the authors' findings, final decisions were referred to one of the senior authors (AÇ).

**Figure 1:**
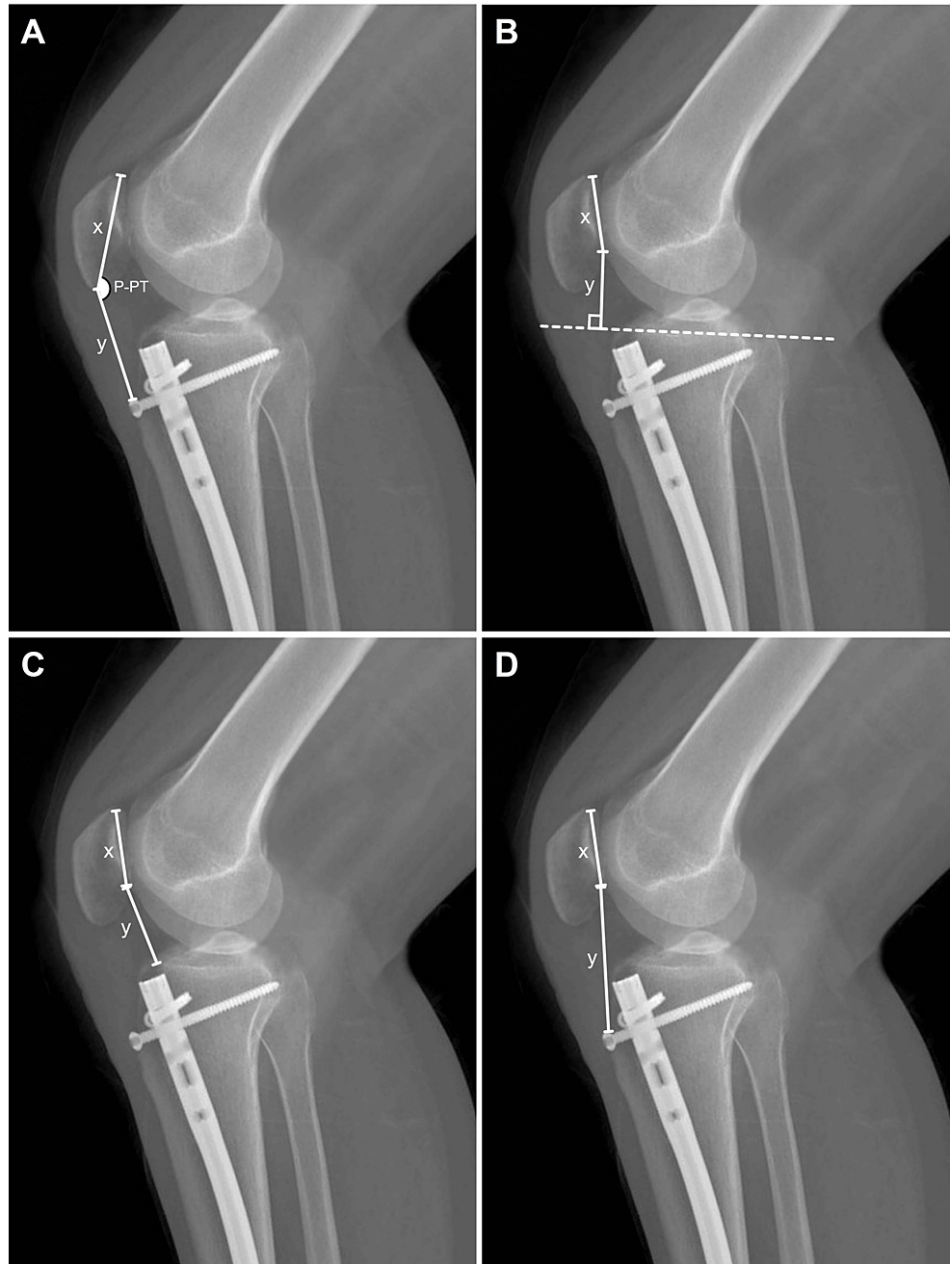
Sample measurements of patellar height indices and sagittal angulation on the lateral direct radiograph of a patient who underwent reamed intramedullary nailing for a tibial shaft fracture: (A) Insall-Salvati index and patella-patellar tendon angle, (B) Blackburne-Peel index, (C) Caton-Deschamps index, and (D) modified Insall-Salvati index.

The Insall-Salvati (IS) index is calculated by dividing the length of the patellar tendon extending from the distal pole of the patella to the tuberositas tibia by the maximum length of the patella extending from its distal to proximal poles [7,8] (Figure 1, Panel A). The calculation of the Blackburne-Peel (BP) index involves dividing the distance between the lower end of the patellar cartilage surface to the tibial plateau perpendicularly by the length of the cartilage surface of the patella [7,8] (Figure 1, Panel B). The Caton-Deschamps (CD) index is obtained by dividing the distance between the lower end of the patellar cartilage surface and the anterosuperior plateau by the cartilage surface length of the patella [7,8] (Figure 1, Panel C). The modified Insall-Salvati (MIS) index represents the proportion of the length of the patellar tendon that extends between the lower end of the patella cartilage surface and the tuberositas tibia to the cartilage surface length of the patella (Figure 1, Panel D) [7,8]. The angle of the patella-patellar tendon (P-PT) is determined by measuring the angle between the line representing the maximum length of the patella, from the distal to the proximal poles of the patella, and the line from the distal pole of the patella to the tuberositas tibia (Figure 1, Panel A) [9,10]. Figure 1 demonstrates the measurements taken on the operated lateral knee radiograph.

Statistical analysis

The statistical analysis was conducted using IBM® SPSS® program version 26.0 (IBM Corp., Armonk, NY). To check for normal distribution conformity, both visual methods (histogram and probability plots) and analytical methods (Kolmogorov-Smirnov test) were utilized. Descriptive statistics were presented as mean and minimum-maximum values for normally distributed variables, median and minimum-maximum values for non-normally distributed variables, and percentage frequency values for categorical data. The paired samples t-test was utilized to compare fractured and intact side values of normally distributed data (IS, CD, and P-PT), whereas the Wilcoxon signed-rank test was employed to compare fracture and intact side values of non-normally distributed data (MIS and BP). Statistical significance was considered significant when the "P" value was less than 0.05.

## Results

Of the 57 patients who underwent tibial IMN surgery at our clinic, 19 patients (33.3%) experienced anterior knee pain, with 15 (78.9%) being male and four (21.1%) females. The average follow-up period was 19.2 months (range: 12-28 months) from the date of measurement. No patients with anterior knee pain displayed any fracture in the proximal third region of their tibia or type 2 open fractures. None of the complex, irregular-shaped fractures were open fractures. No intraoperative observation of patellar tendon injury or rupture occurred in any patients. Table 1 shows the additional patient demographics.

**Table 1:** Demographic profile of the patients N: Number of patients. * Median and minimum-maximum ranges were used as descriptive statistics. ** Mean and minimum-maximum ranges were used as descriptive statistics.

		Patients (%) (N = 19)
Age* (years)		30 (Range: 18–74 years)
Follow-up** (month)		19.2 (Range:12–28 months)
Gender	Female	4 (21.1%)
	Male	15 (78.9%)
Side	Right	9 (47.4%)
	Left	10 (52.6%)
Injury mechanism	Simple fall	6 (31.6%)
	Fall from height	4 (21.1%)
	Traffic accident	9 (47.4%)
Gustilo-Anderson open fracture type	None	9 (47.4%)
	Type 1	10 (52.6%)
	Type 2	0
Fracture location	Distal 1/3	14 (73.7%)
	Middle 1/3	5 (26.3%)
Fracture geometry	Spiral	9 (47.4%)
	Transverse	6 (31.6%)
	Complex-irregular	4 (21.1%)

No statistically significant differences were observed in patellar height indices (IS, BP, CD, and MIS) between the fractured and intact sides of the patients (p = 0.588; p = 0.747; p = 0.446; p = 0.573, respectively). However, a statistically significant difference was found in P-PT angles between the fractured and intact sides of the patients when evaluated in terms of sagittal angulation (p = 0.048) (Table 2).

**Table 2:** Postoperative patellar height indices and patella-patellar tendon angle changes in fractured and intact knees of patients with anterior knee pain P: Statistical significance value. Patient group: Measurements made on the operated extremities of patients with anterior knee pain after tibial intramedullary nailing. Control group: Measurements made on the intact extremities of patients with anterior knee pain after tibial intramedullary nailing. * Mean and minimum-maximum range values were used as descriptive statistics. ** Median and minimum-maximum range values were used as descriptive statistics.

	Patient group	Control group	P-values
Insall-Salvati index*	0.901 (Range: 0.726–1.119)	0.887 (Range: 0.712–1.028)	0.588
Blackburne-Peel index**	0.890 (Range: 0.856–1.8)	1.341 (Range: 0.772–1.628)	0.747
Caton-Deschamps index*	0.993 (Range: 0.792–1.271)	0.942 (Range:0.415–1.376)	0.446
Modified Insall-Salvati index**	0.539 (Range: 0.361–0.706)	0.594 (Range: 0.415–0.662)	0.573
Patella-Patellar tendon angle*	145.53° (Range: 136°– 160°)	142.89° (Range: 134°–161°)	0.048

## Discussion

Anterior knee pain after tibial IMN is a common postoperative complication that significantly affects patients' quality of life [2,3,5]. Identifying, managing, and even preventing the underlying factors that contribute to this complication can notably improve the patient's comfort and postoperative quality of life. Various studies in the literature have investigated anterior knee pain [2,3,9]. However, our knowledge suggests that research examining the correlation between patellar height and anterior knee pain following tibial IMN is severely limited. This represents the primary strength of our study. Our research revealed that there is no significant link between patellar height and anterior knee pain after undergoing tibial intramedullary nailing with a reamed tibia IMN alongside an infrapatellar patellar tendon split approach (p < 0.05). A noteworthy discovery was the significant correlation between the P-PT angle used for measuring sagittal angulation and anterior knee pain that occurs after tibia IMN (p = 0.048).

Anterior knee pain following IMN is a common complication, with a prevalence ranging from 30% to 50% according to existing literature [2,3,5]. Obremsky et al. reported a 48% incidence of anterior knee pain following IMN in their study [17]. Similarly, Erinç et al. found that 46.2% of patients experienced this pain in their local study [18]. In our study, only 19 out of 57 patients who underwent tibial IMN reported anterior knee pain, accounting for 33.3% of the sample population. Although all the patients underwent infrapatellar patellar tendon split nailing, our complication rate was comparatively lower than that reported in the literature. This may be due to differences in the definition of complications. Specifically, most of the previous studies included patients who complained of anterior knee pain during at least one outpatient clinic visit [2,3,9]. In contrast, our study involved patients who continued to report anterior knee pain in at least two outpatient clinic visits, which were one month apart during the follow-up postoperation. The aim of our research was to remove any immediate unease that could be misconstrued as anterior knee pain, for example, annoyance caused by the suture material utilized on the patellar tendon, and consequently establish the impact of patella alignment on pain, in an unbiased manner.

Patella alta poses a considerable risk for patellofemoral instability [19,20], which is a leading cause of anterior knee pain [21]. Anterior knee pain is also frequently associated with patellar malalignment and extensor mechanism issues, both of which are related to patellar height [7,8,21]. Based on our hypothesis, our study investigated the difference in patellar height values between patients with anterior knee pain who underwent tibial intramedullary nailing and the control group. However, our findings do not support our hypothesis. We found no significant difference in patellar height values across all evaluated indices between the patient and control groups (p > 0.05). To further elucidate this discovery, it is worth noting that patellar balance is intricately linked to the strength and balance of both the quadriceps and hamstring muscles [22,23]. While all patients in our study underwent the same rehabilitation process to attain uniform muscular strength, more comprehensive findings can be achieved through the objective measurement of quadriceps muscle strength and its comparison with patellar height index values in future investigations. It is important to take into account the intricate structure of the knee and the various relationships between ligaments and tendons, which affect its motion. As magnetic resonance imaging was not used in our study, we had no information about the ligament balances in the patients' knees. Additionally, it is worth mentioning that anatomical differences in the patella or patellar articular surface could affect all measurements of the patellar height index. Finally, the small number of patients involved in our study might have influenced our results.

A statistically significant decrease in the postoperative P-PT angle was observed in our study (p = 0.048), resulting in an approximate three-degree change. The literature indicates that even slight declines in this angle value hold clinical significance due to the association between patellar tendon strain, imbalanced load anterior to the knee, and tendinopathy with P-PT angle values [9,10,23,24]. The decrease in P-PT angle post-surgery in tibial IMN may suggest a decrease in anterior knee loads or the effect of the patellar tendon split technique on the patellar tendon. Nevertheless, it is essential to note that the location of the patella in the sagittal plane is mainly determined by the quadriceps muscle [9,10,21,22]. Previous literature has shown that the patellar sagittal balance may be impacted by the seesaw effect caused by weakness in the quadriceps [9]. Notably, our study is limited in its comprehension of the cause of the P-PT angle adjustment as there is a lack of objective measurements of muscular strength in the quadriceps muscle group. Large patient cohorts, together with comprehensive clinical, biomechanical, and cadaveric studies, could reveal the kinematics and relationship of anterior knee pain following tibia IMN.

Our study has various limitations. First, the control group used was the opposite knee of the patients. While literature reports anatomical similarities of both sides [6,16], obtaining optimum results requires measurements of patellar height and sagittal angulation from a control group of patients with no prior anterior knee pain after tibial IMN. Second, the relatively low number of patients is a significant limitation. Finally, it is important to note that our analysis is exclusively based on radiologic measurements. Although our study aimed to demonstrate the effect of patellar height on anterior knee pain, broader results can be obtained through studies that address multiple factors, such as muscle strength and range of motion, and employ multifaceted analyses with large cohorts of patients.

## Conclusions

Anterior knee pain is a common postoperative complication after IMN of the tibia, significantly impacting patients' quality of life and comfort. Our research found no conclusive evidence demonstrating the correlation between patellar height and anterior knee pain following reamed IMN of the tibia using the infrapatellar patellar tendon split technique. Nonetheless, we detected a notable relationship between alterations in sagittal angulation and anterior knee pain. Patellar sagittal balance has been identified as one of the contributing factors to the development of anterior knee pain following reamed tibial IMN. Further biomechanical and comprehensive clinical studies are necessary to fully investigate the correlation between the three-degree increase in sagittal patellar angulation and the occurrence of anterior knee pain.
